# Spatial configuration of cultural landscape facilities in Shandong’s Yellow River corridor: a rural living circle perspective

**DOI:** 10.3389/fpubh.2026.1832336

**Published:** 2026-05-08

**Authors:** Zhiwei Zhang, Wen Li, Wei Liu, Ye Huang, Yiqun Xu

**Affiliations:** 1 School of Architecture and Urban Planning, Shandong Jianzhu University, Jinan, China; 2 Jinan City Planning and Design Institute, Jinan, China

**Keywords:** accessibility, cultural landscape facilities, mobile signaling data, point-of-interest (POI) data, rural living circle

## Abstract

The rural cultural landscapes in the Yellow River Basin, as an important part of the blue-green infrastructure, not only provide ecological services and public spaces, but also carry the function of regional cultural inheritance. The spatial layout of rural cultural landscapes deeply aligns with the multi-level living circles of residents, and has a significant impact on the organization of landscape spaces and social ecological resilience in rural areas. This study took 14,502 administrative villages in 25 counties (cities and districts) along the Yellow River in Shandong Province as the research objects. Based on mobile phone signaling data, the boundaries of rural living circles at the three scales of towns, counties and cities were identified, and the accessibility evaluation indicators of six types of cultural landscape facilities were constructed in combination with POI data. The aim is to reveal the service level of rural cultural landscapes and their potential role in promoting health equity and resilience. The research shows: (1) The travel radius of rural living circles in the Yellow River region as a whole shows an increasing trend from upstream to downstream, with a concentric expansion feature centered on county towns. The average radius of township, county and municipal living circles is approximately 8 km, 13 km and 18 km respectively; (2) The overall accessibility of cultural landscape facilities shows a multi-center and ring-shaped distribution pattern. There are significant spatial differences in the spatial distribution of different types of facilities. Among them, landscape green spaces form a high accessibility zone with a relatively high degree of continuity relying on prefecture-level cities and county parks. Sports, leisure, science and education, and cultural facilities are concentratedly distributed in county towns and key towns. Cultural relics, ancient sites and scenic spots are scattered in a point-like manner due to historical evolution and natural conditions. The cultural facilities along the main stream of the Yellow River are arranged in a string-like pattern; (3) The results of geographically weighted regression (GWR) show that the effects of different facility types on the characteristics of living circles have significant spatial non-stationarity. The response degree in the middle section of the Yellow River region is relatively higher. The research results can provide quantitative support for the layout optimization of rural cultural landscape facilities and the construction of living circle systems in the Yellow River region.

## 1 Introduction

The Yellow River is the mother river of the Chinese nation and an important carrier of cultural identity. The Outline of the Plan for Ecological Protection and High-quality Development of the Yellow River Basin emphasizes that “efforts should be made to protect, inherit and carry forward the Yellow River culture, and make the Yellow River a happy river for the benefit of the people”, and proposes to “systematically protect, deeply inherit, tell good stories, and create a cultural tourism belt”, which clearly elevates culture as one of the core tasks of basin governance and regional development (1). In this context, cultural landscape facilities in this study refer to publicly accessible spatial carriers rooted in natural, historical and cultural resources that simultaneously provide ecological, recreational, educational and heritage services (2, 3). The Yellow River culture originated in the countryside and flourished in rural areas. The rural cultural landscape facilities are rooted in the local natural and humanistic foundation and lifestyle. Through systematic organization, they transform the memories, production, and daily activities of rural culture into a facility system that continuously provides public services and landscape experiences (4). The rural cultural landscape facilities along the Yellow River not only include various types such as public green spaces, sports and leisure areas, science and education culture, historical relics, and scenic spots, but also form a multi-level system embedded with the Yellow River-themed culture. They not only serve the current production and living needs, but also carry the dynamic inheritance of the history of river management and local culture (5, 6). In the rural areas along the Yellow River, the “living circle” serves as a spatial unit that organizes villagers’ daily production and living behaviors. It constitutes the social space carrier for the precise implementation, fair allocation, and coordinated operation of the “green and blue climate adaptation and health equity infrastructure”. Specifically, blue and green infrastructure (such as river-side wetlands, ecological revetments, rainwater storage green spaces, ventilation corridors, community cold spots, etc.) are based on the living circle as the basic planning and assessment unit. They can embed climate adaptation goals (such as alleviating heat waves, flooding, and soil erosion) and health equity demands (such as ensuring that the older adults, children, the sick, and the disabled have equal access to cooling, clean water, rest, and emergency shelters) into the daily paths that villagers can walk to. Conversely, if the perspective of the living circle is not considered, the blue and green infrastructure is likely to become fragmented or elitist landscape projects, which are difficult to respond to the significant differences in flood control pressure, aging levels, and accessibility of public services among different villages along the Yellow River, thereby exacerbating climate vulnerability and health inequality.

The rural living circle takes the administrative village-natural village as the basic service unit, and arranges basic public services according to the daily activity radius and service accessibility of residents, forming a multi-level spatial unit, and translating dispersed resources into accessible, participatory and sustainable daily experiences. Within this framework, the living circle defines the spatial boundary, travel threshold and service hierarchy of residents’ daily activities, while cultural landscape facilities constitute one of the key facility systems organized within this boundary. Therefore, the living circle provides the behavioral-spatial scale for evaluating whether cultural landscape facilities are reachable, hierarchical and matched to daily needs. The research based on living circle has important guiding significance for urban and rural planning practice (7). The characterization of structure circle and function circle can directly guide the scale, type and layout of facilities configuration (8, 9). As an important issue in the fields of social geography and transportation geography, the study on the characteristics of rural living circle has substantial guiding value for rural construction and governance practice. Current research mainly focuses on the following aspects: (1)Identification of zoning layers and their formation mechanisms, focusing on the hierarchical construction and spatial boundary delineation of structural zoning layers and functional zoning layers, and explaining the formation and variation of living zones from the perspective of individual/family daily behaviors and time–space budget (7); (2)Research on accessibility of facilities and configuration optimization, under the conditions of multi-modal travel and time–space constraints, measuring accessibility and service radius, and then conducting model-based optimization of facility-population matching and location/ capacity to achieve a balance between fairness and efficiency (10); (3)Study on scenario flexibility, incorporating time-varying scenarios such as busy/fair seasons, festivals/daily life, and flood/drought periods into the assessment, and identifying the seasonal flexibility of living zones (11); (4)Research on corridor regions and cross-village collaboration, in corridor-type regions such as rivers, emphasizing the network collaboration of “nodes—corridors—settlement surfaces” and cross-village sharing and corridor coordination (12).

Current research generally indicates that the spatial structure of the living area has a strong coupling relationship with the configuration of public facilities: the former defines the service radius and the boundary of the area through the distribution of residences, travel methods and activity chains, while the latter determines the scale, hierarchy and layout of the facilities (8). Empirical research further indicates that there are significant differences among groups such as the older adults (9, 31), school-aged children (13), women (14), and vulnerable groups (15) in terms of travel capacity, time budget, and arrival threshold. Therefore, a stratified and graded, accessible allocation strategy is needed (16). In rural settings, this demand becomes even more prominent. The scattered population, strong seasonal variations, and sparse public transportation make it necessary for the living area—facility configuration—to strike a balance between fairness and efficiency, and to align with local social networks and daily practices (17). Rural cultural landscape is embedded in residents’ daily behavior and place use. How to make cultural landscape facilities and rural living circle achieve effective coordination, which not only serves the current production-living, but also carries the live inheritance of local culture, has become a key issue that needs to be responded to in the construction of rural living circle.

In view of this, based on the functional circle theory of living circle, this paper uses POI data and mobile phone signaling data to identify the scope of living circle at different levels, explore the accessibility level characteristics of rural living circle at different levels, analyze the quantitative relationship between the spatial scope of rural living circle and all kinds of cultural landscape facilities, and analyze the characterization of this relationship at different spatial scales.

## 2 Research methods and data sources

### 2.1 Research scope

**Figure 1:**
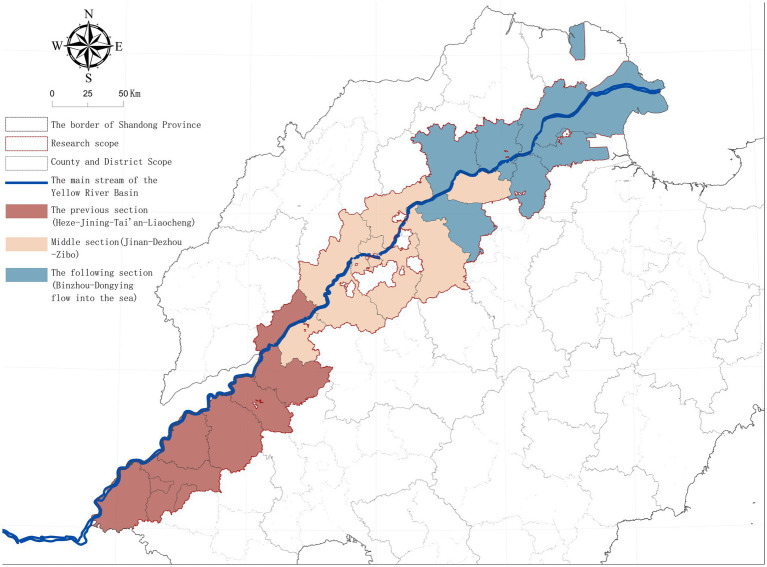
The research area location map.

The main stream of the Yellow River flows through nine prefecture-level cities in Shandong Province, namely Heze, Jining, Tai’an, Liaocheng, Jinan, Dezhou, Zibo, Binzhou and Dongying, covering 25 counties (cities and districts). Its macro spatial form is typically in a band-like distribution (Figure 1). It is adjacent to the Hai River Basin in the north and connects with the Huai River Basin in the south, forming a relatively unique regional-cultural interface. This study takes the county-level areas along the Yellow River in Shandong Province as the research scope, with the movement of rural residents as the research object. After excluding the urban built-up areas, a total of 14,502 villages were included, with a research area of approximately 25,747 km^2^. To facilitate the identification of different hydrological conditions and the differences with the cultural lineage, the research area is divided into the upper section (Heze—Jining—Tai’an—Liaocheng), the middle section (Jinan—Dezhou—Zibo), and the lower section (Binzhou—Dongying to the sea).

### 2.2 Data source

This study mainly includes two types of data: (1) mobile signaling data. The data is from a certain operator and contains travel trajectory information. The spatio-temporal information is marked with high precision and strong continuity, which can accurately reflect the real travel connections. The data period is May 2022, with a time interval of 30 min and a spatial accuracy of 500 m × 500 m geographic grid. After data cleaning and outlier processing, a total of 5,592,676 daily life travel chain records were obtained in the study area, including the starting point geographic grid number and its geographic coordinates, the ending point geographic grid number and its geographic coordinates, and the number of residents corresponding to each starting point geographic grid, which provided basic support for identifying residents’ travel connections and living circle levels. (2)Point of Interest (POI) data. It comes from Google Maps in 2024. The original data covers 20 primary categories, and each primary category contains several medium and small categories. Each POI contains attribute information such as longitude, latitude, name, address, type, and administrative region. According to the research needs, six types of facilities were extracted: landscape green spaces, sports and leisure services, scientific and cultural services, cultural relics and historic sites, yellow river related cultural facilities, and scenic spots. A total of 13,336 POI locations were obtained, and based on this, 93 cultural facilities related to the Yellow River were identified (Table 1).

**Table 1:** Classification of cultural landscape facilities.

Facility classification	Main containment types
Landscape green spaces	City squares, parks and park squares, zoos, botanical gardens, aquariums and their supporting service facilities
Sports and leisure services	Sports and leisure service venues, vacation and recuperation places, golf-related areas, parks and squares, shopping malls, theaters, entertainment venues, sports stadiums
Scientific and cultural services	Museums, urban squares for educational, cultural and recreational services, archives, science and technology museums, art galleries, libraries, cultural centers, performing arts groups, exhibition halls, and related educational and cultural services of government agencies.
Cultural relics and historic sites	Islamic temple, memorial hall, church, World Heritage Site, temple and Taoist temple
Scenic spots	Scenic spots and resorts, viewing points, national-level scenic spots, national-level scenic spot companies and enterprises, tourist attractions, provincial-level scenic spots, township-level scenic spots
Yellow river related cultural facilities	—

The administrative villages in the rural areas along the Yellow River in Shandong Province have significant differences in their village domain areas. However, the built-up radius of typical administrative villages is usually 300–800 m. According to the field research data, the interquartile range of the built-up area of administrative villages is 0.18–0.68 km^2^. The 500 m × 500 m grid can well approximate the spatial scale of administrative villages. In addition, the 500 m grid is a commonly used size when commercial signaling data providers perform spatial aggregation in rural areas. This size can effectively suppress individual-level location noise while retaining the information of inter-village travel connections, and at the same time meet the basic requirements of data de-identification and privacy protection. Considering that there might be a problem of spatial resolution mismatch in the data, we, based on the actual situation, finally unified all the data for analysis at a resolution of 500 m.

### 2.3 Research methods

**Figure 2:**
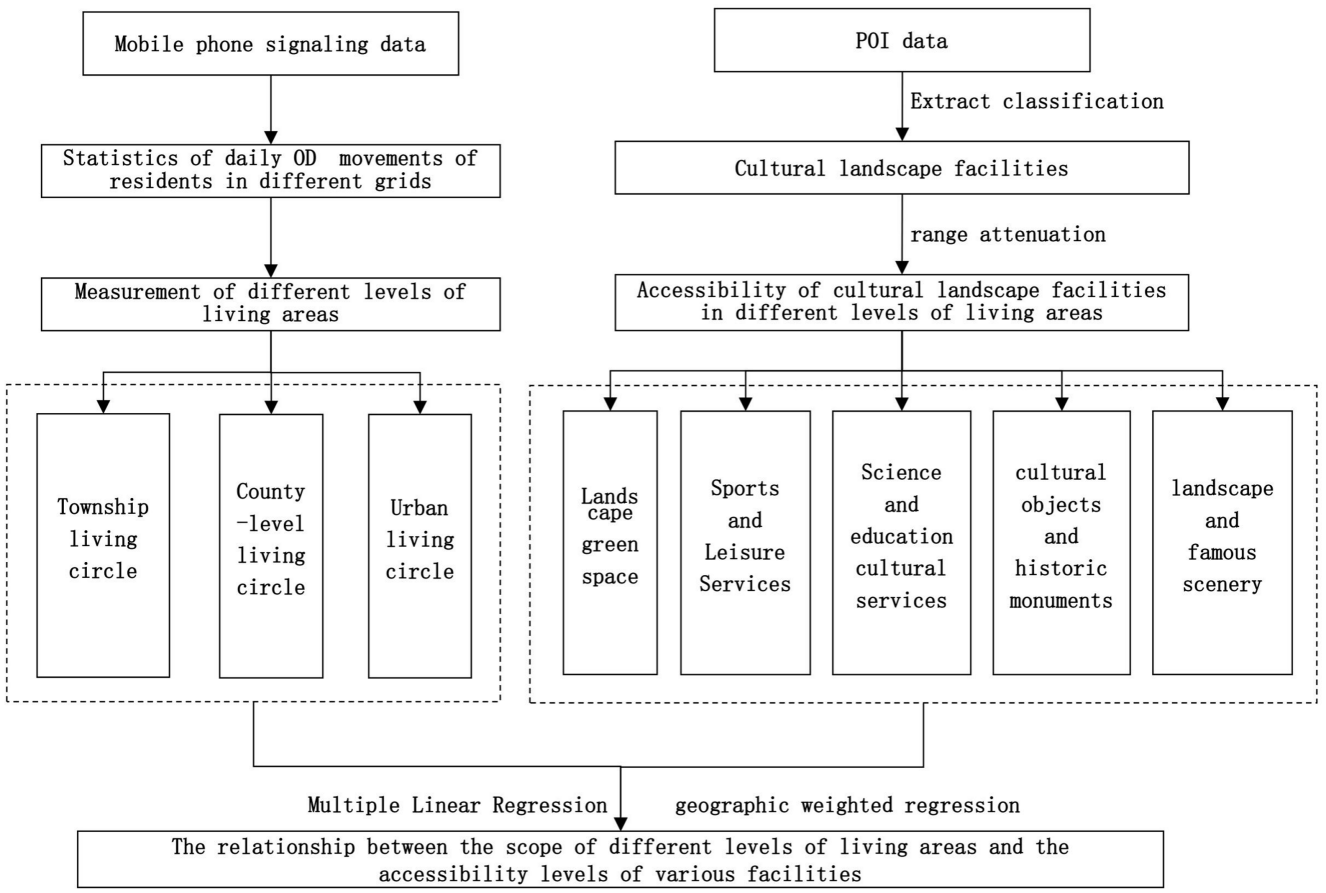
Research method flowchart.

Firstly, based on mobile phone signaling data, the different levels of living circle radii of rural residents within each geographical grid are identified. Secondly, based on the POI data of cultural landscape facilities, the accessibility of different cultural landscape facilities to rural residents within each geographical grid is calculated. Finally, using multiple linear regression and geographically weighted regression models, the relationship between different levels of living circles and the accessibility levels of cultural landscape facilities is analyzed (Figure 2). For the facility classification process, based on the facility names, geographical coordinates and address texts for spatial and semantic matching, overlapping records are identified. When the same facility has multiple attributes, it is classified into the most core and irreplaceable category in rural cultural inheritance and daily life. Meanwhile, we also adopted manual review to make a final ruling on the facilities still in doubt in the automatic classification results by combining the field research photos and the actual situation of satellite remote sensing, so as to ensure the mutual exclusiveness of the six types of facilities in spatial statistics, and at the same time retain the complexity of cross-category facilities in the subsequent neighborhood analysis.

#### 2.3.1 Measurement of living circle

Firstly, the travel radius of rural residents along the Yellow River is quantitatively identified using mobile phone signaling OD data. After preprocessing such as eliminating outliers and restricting to the study area, each village was used as the starting unit to measure the daily commuting distance distribution of residents. The specific method is as follows: taking village *i* as the starting point, all its commuting OD are sorted by travel distance from near to far, and the number of trips in each record is recorded as $w_{\mathrm{ij}}$ and the distance is $d_{\mathrm{ij}}$ . The commuting records within the same village are accumulated to obtain cumulative travel numbers $W_{\mathrm{ij}}$ and total travel numbers $W_{i}$ , and the corresponding cumulative travel rate is calculated using Equation (1):


Pij=WijWi (1)

On this basis, the travel distance corresponding to cumulative travel rates from 10 to 90% (at 10% intervals) was extracted for each origin village and defined as the circle radius under different cumulative travel levels. To reflect the overall travel structure of the study area, the final cumulative travel rate-radius curve used for boundary identification was constructed by weighting each origin by its total trip volume. The equal-weighted curve is retained only as a robustness comparison and is not used to determine the final living-circle thresholds.

**Figure 3:**
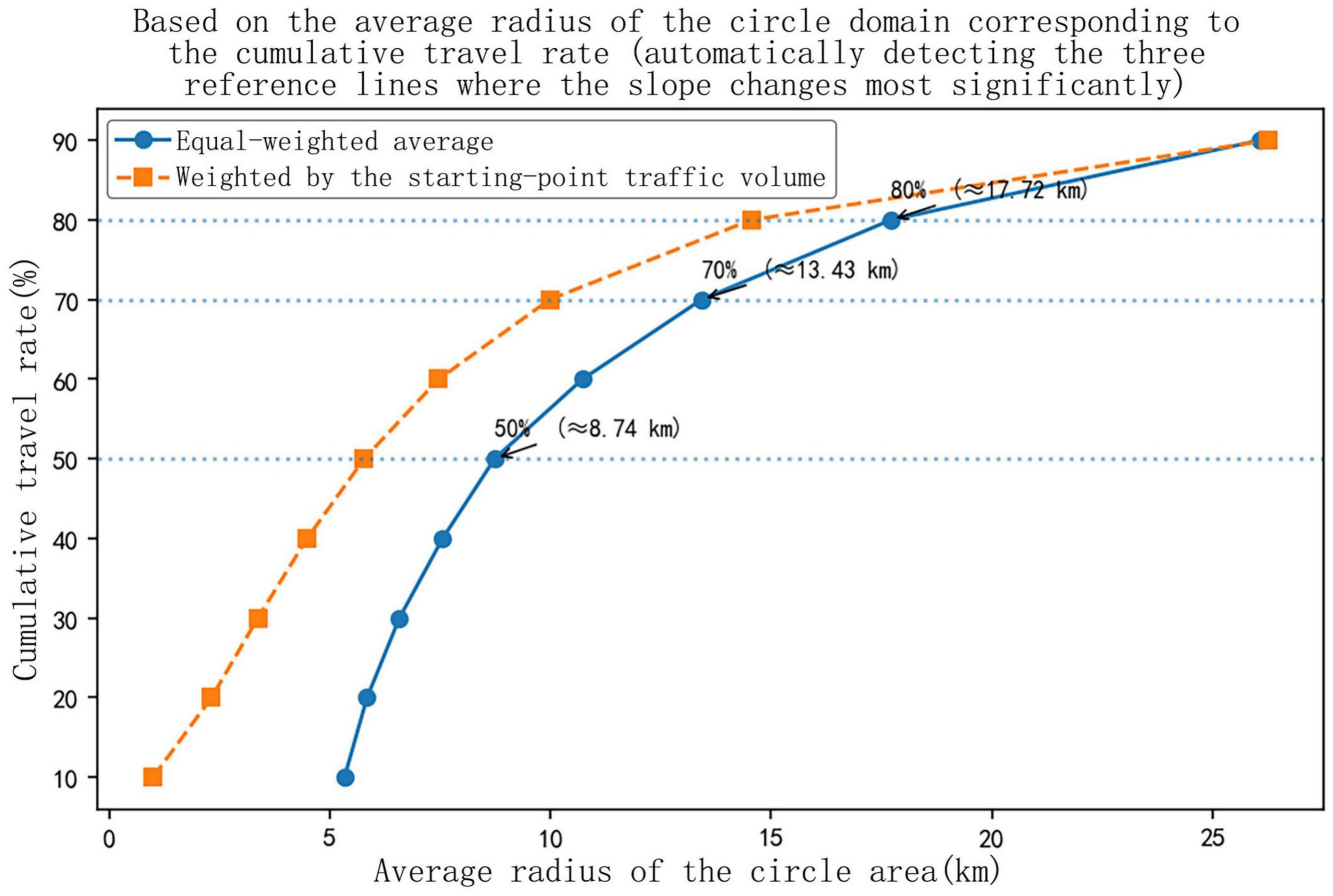
Diagram showing the radius division of the social circle layers.

As shown in Figure 3, the trip-volume-weighted cumulative travel rate-radius curve exhibits evident slope transitions around 50, 70, and 80%, indicating three relatively stable travel layers from near to far. In this study, these values are treated as empirical thresholds derived from the observed travel structure rather than as universal normative cutoffs. Their interpretation is further informed by the hierarchical logic commonly discussed in living-circle and public-service planning, in which nearby space tends to support high-frequency daily needs, intermediate space corresponds more to county-oriented medium-frequency trips, and outer space captures lower-frequency city-oriented activities (11, 18–20). Accordingly, the township living circle is defined as the radius of 8.74 km corresponding to the 50% cumulative travel rate, reflecting the nearby and high-frequency activity space for daily shopping, errands, and basic cultural leisure. The county-level living circle is defined as the radius of 13.43 km corresponding to the 70% cumulative travel rate, representing medium-frequency and medium-distance trips that mainly connect residents to higher-order education, medical, and comprehensive public services within the county. The urban living circle is defined as the radius of 17.72 km corresponding to the 80% cumulative travel rate, reflecting lower-frequency and longer-distance trips oriented to prefecture-level urban services and major Yellow River cultural nodes. Together, the three circles describe a progressive relationship between activity frequency, service hierarchy, and spatial reach from the inside out (11, 19, 20).

#### 2.3.2 Accessibility measures of cultural landscape facilities

The accessibility of cultural landscape facilities is measured using the distance attenuation method based on the shortest distance (21). The research area covers the rural areas along the Yellow River in Shandong Province. The road network data is largely incomplete or poorly recorded in areas such as floodplains and the back slopes of dams (such as unpaved production roads and seasonal waterlogged roads). If the road network distance is forcibly used, a large number of hypothetical completions of the missing sections are required, which may introduce greater errors. Therefore, we adopted the Euclidean straight-line distance in this context, as it provides a unified, reproducible and unbiased benchmark. Taking the centroid of each village as the spatial position of the residents, the villages and six types of cultural landscape POI facilities are uniformly projected into the metric coordinate system. Then, the straight-line distance from each village to the same type of POI is calculated. In this study, i represents the village, s represents the facility type, and $d_{\mathrm{ij}}$ represents the distance between village i and the jth facility of this type. The shortest distance from village i to the s-type facilities is recorded as Equation (2):


$$d_{i,s}^{\min}=\min(d_{\mathrm{ij}}),j=1,2,3\ldots \ldots n_{s}$$ (2)

In the formula, $n_{s}$ represents the number of facilities of type s. Considering that residents’ travel has a distance-decaying characteristic, the closest distance is regarded as the travel impedance, and the accessibility index is constructed as follows:


$${A_{i,s}}^{=}\frac{1}{d_{i,s}^{\text{minβ}}}$$ (3)

In Equation 3, $A_{i,s}$ denotes the accessibility index of village i to facility type s, and *β* is the distance-decay coefficient. Based on relevant studies and the travel characteristics of residents in the study area, *β* is set to 1. Since $A_{i,s}$ is inversely related to ${d_{i,s}^{\min}}^{\beta }$ , accessibility decreases monotonically as the nearest distance increases. Therefore, in the subsequent spatial mapping, the classified values of ${d_{i,s}^{\min}}^{\beta }$ are used to represent accessibility levels, with shorter nearest distances indicating higher accessibility.

#### 2.3.3 Multiple linear regression models

To analyze the relationship between different levels of living areas and the spatial configuration of cultural landscape facilities, this paper constructs multiple linear regression models at three different scale levels: township travel area, county travel area, and municipal travel area. The radius of each village’s area is taken as the dependent variable, and the accessibility indicators of six types of cultural landscape facilities are used as independent variables, including landscape green space type, sports and leisure service type, science and education culture type, cultural relics and ancient sites type, scenic spot type, and the nearest distance or standardized accessibility value of Yellow River cultural facilities. In addition to the six accessibility variables of cultural landscape facilities, three control variables were included in the regression models, namely population size, distance to the county seat, and distance to the township center. These variables were introduced to control for differences in settlement scale and locational conditions that may affect the radius of rural living circles. Ordinary least squares method (OLS) is used for parameter estimation, and the model fitting effect is evaluated through significance tests and determination coefficients and other indicators.

#### 2.3.4 Geographically weighted regression

Geographically Weighted Regression (GWR) is used to analyze “whether the regression relationship is consistent spatially”. As a local modeling approach, GWR explicitly captures spatial non-stationarity and allows regression coefficients to vary across space (22, 23). This study explicitly incorporates spatial heterogeneity on the basis of the traditional Ordinary Least Squares (OLS). Unlike the global model that only estimates a set of overall regression coefficients, GWR assumes that the influence coefficient of the independent variable on the dependent variable can vary with the spatial location in each geographical unit. During the estimation process, a spatial weight matrix is constructed through a kernel function, giving higher weights to sample points near the target unit and lower weights to those far away. This study uses the Gaussian kernel function and utilizes the Akaike Information Criterion (AIC) or its modified form (AICc) to automatically select the optimal bandwidth, in order to achieve a balance between overall fit and local stability. The specific approach is: on the same setting of dependent and independent variables as in multiple linear regression, GWR models are constructed for different living zones (townships, counties, and municipal areas), and the local regression coefficients and corresponding t-values of various cultural landscape facilities are spatially visualized to identify which facilities and where in different regions along the Yellow River have significant promoting or inhibiting effects on the living circle scale.

## 3 Results

### 3.1 Measurement of accessibility of rural living areas and cultural landscape facilities

#### 3.1.1 Identification of living circle types

**Figure 4:**
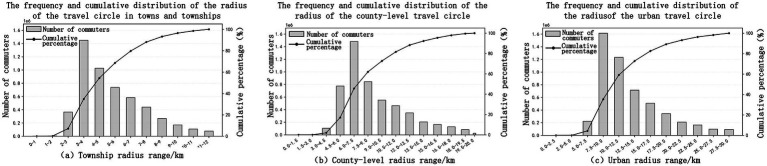
Characteristics of different levels of living circles.

The spatial distribution of the residential living area exhibits distinct hierarchical characteristics as shown in Figure 4. In the township living circle, the number of people in the travel radius of 3–4 km is the largest, which is the core dense area of the whole living circle. After 4 km, the scale of travel population begins to decline, and about 90% of the population is concentrated within 9 km from the center, indicating that the core service range of the township living circle is within 9 km (Figure 4a). In the county-level living area, a high value band is formed within the 4.5–9 km range, with the peak occurring at 6–7.5 km. Approximately 80% of the population is concentrated within 10 km, and reaches 90% around 15 km, indicating that the effective service radius of the county-level living area is approximately 15 km (Figure 4b); In the metropolitan area living area, the frequency of traveling population is higher within the 7.5–12.5 km range, and significantly decreases after 17.5 km. By around 20 km, the cumulative proportion has approached 90%, indicating that the main service range of the metropolitan area living area is within 20 km, and 20–30 km is the expansion area of the living area (Figure 4c).

#### 3.1.2 Spatial differentiation of different levels of living areas

**Figure 5:**
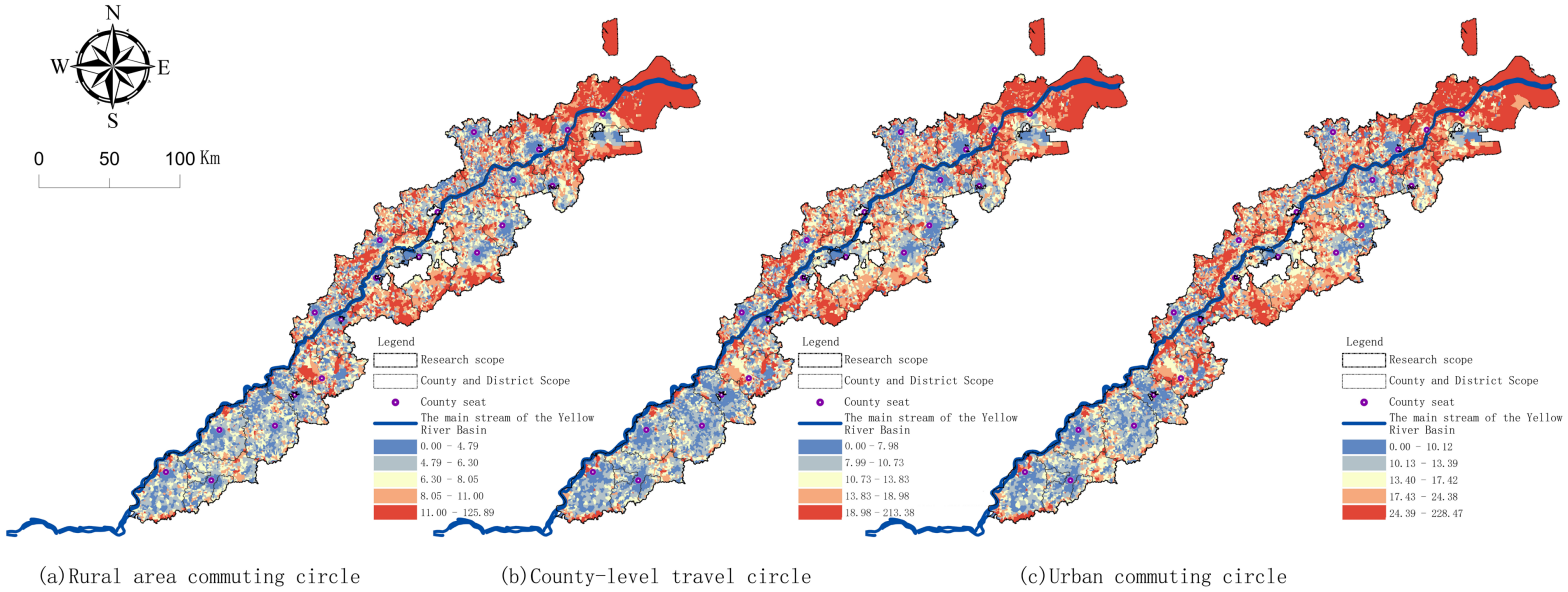
Spatial distribution of the radii of different levels of living circles: **(a)** township living circle; **(b)** county-level living circle; **(c)** urban living circle.

The living circles of towns, counties and cities along the Yellow River in Shandong province show a spatial pattern of overall increase from upstream to downstream and circle expansion around county residence (Figure. 5). (1) At the level of township living circle, the overall travel distance is relatively short, and the low-radius patches with radius ≤ 8 km are formed near the county resident and the river channel. in the downstream area of Binzhou-Dongying tidal flats, due to sparse river-crossing channels and water system division, a continuous high-radius zone-like area is formed in the periphery (Figure 5a). (2) At the county living circle level, the medium and high value travel increases significantly, and the radius increases from the county resident as the core to the outside; travel distances in the upstream are significantly lower than those in the middle and lower reaches (Figure 5b). (3) At the level of urban living circle, the distribution of high values is further expanded. Except for a relatively low value band along the yellow main trunk and the upstream region, the middle, downstream and outer coastal edge are mostly orange-red long-distance areas, indicating that long-distance travel has become a dominant feature at the municipal scale (Figure 5c).

#### 3.1.3 Accessibility of cultural landscape facilities

**Figure 6:**
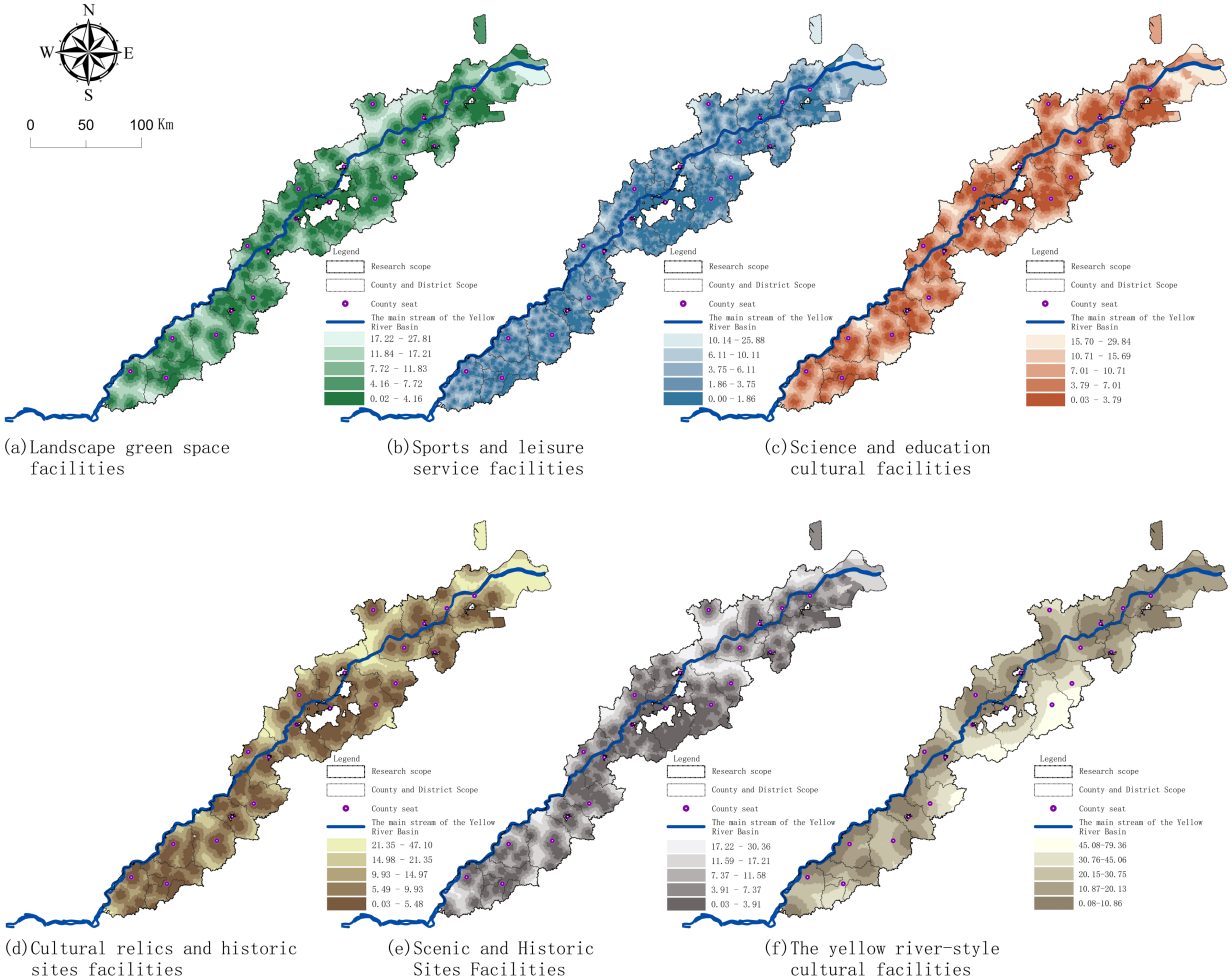
Spatial distribution of accessibility for various cultural landscape facilities: **(a)** landscape green space facilities; **(b)** sports and leisure service facilities; **(c)** science and education cultural facilities; **(d)** cultural relics and historic sites facilities; **(e)** scenic spots facilities; **(f)** Yellow River-related cultural facilities.

The accessibility levels of cultural landscape facilities all exhibit a multi-center and ring-shaped concentric structure. Spatially, the middle-eastern section is superior to the western section, and the upper-middle reaches are superior to the lower reaches, which is basically consistent with the distribution of urban density. There were also significant differences in the accessibility of different types of cultural landscape facilities: (1) The landscape green space category was centered on the green space system of prefecty-level cities and county parks, forming a high accessibility ring belt with higher continuity and slower attenuation, which radiated the most stable radiation to surrounding villages. (2) Sports leisure services are highly concentrated in county seats and key towns and villages, and the decline is fastest towards the outskirts. The boundary of accessibility layer is clear, and the continuous high value feature in the Middle East is prominent. (3) The accessibility gap between science, education and culture towns and villages is significant, and the high value areas are basically coincident with the county resident. (4) Restricted by the distribution of historical remains, the cultural relics and historic sites clustered in a point-sheet pattern, with high value patches concentrated in the upstream and steep attenuation to the periphery, and the overall continuity was weak. (5) The scenic spots are dominated by natural resource endowments, forming a double-high value area of “Taishan forebelt—estuarine coastal belt”, and the middle reaches of the corridor are relatively loosely connected, with discrete patches. (6) The cultural facilities of the Yellow River show a beaded node layout along the main stream, and the corridor attribute is the most prominent. The continuity of the middle and lower reaches is stronger than that of the upper reaches. However, the coverage of the inner cities is limited, and the cultural facilities mainly spread along the river to both sides (Figure 6).

### 3.2 The influence of cultural landscape facility accessibility on the radius of the living area

#### 3.2.1 Multivariate linear regression results

From the model fitting results shown in Table 2, all three scales of the models passed the significance test, indicating that there is indeed a stable relationship between the radius of the living circle and the accessibility of various cultural facilities. Specifically: (1) The accessibility of cultural facilities related to the Yellow River shows a significant negative trend across all three scales, and the effect increases with the expansion of the scale (township—0.007**, county—0.016***, city—0.035***). This indicates that the more Yellow River cultural facilities there are and the easier they are to reach, the more the activity range is concentrated in the local area, especially at the city scale. (2) The accessibility of cultural relics and historic sites, science, education, culture and sports and leisure facilities are significantly positive in the three scales, which indicates that these three types mostly belong to facilities with higher grades and stronger purpose. This phenomenon is more obvious in a large range, especially for cultural relics and historic sites. (3) The facilities of scenic spots are significantly negative at the township and county scale (township −0.069***, county −0.067***), but not significant at the city scale, indicating that at the township and county scale, people are more willing to visit the nearby scenic spots, thereby shortening the average travel distance; however, when expanded to the city scale, both short-distance and long-distance tourism exist simultaneously, and the effects cancel each other out, resulting in no significant effect. (4) Landscape green space facilities are positive (0.028**) at the township scale, insignificant at the county level, and negative (−0.065**) at the city level, which means that the improvement of the accessibility of green space at the township level will cause some additional travel, and then expand the living circle slightly. While at the city scale, relatively complete urban green space supply disperses long-distance leisure needs over a larger area, overall shortening the average travel distance for residents.

**Table 2:** The results of the multiple linear regression model for the radius of living areas and the accessibility level of various facilities.

Types	Township living circle		County living circle		Metropolitan area living circle	
	Standardized regression coefficient	*t*-value	Standardized regression coefficient	*t*-value	Standardized regression coefficient	*t*-value
Landscape green spaces	0.028**	2.398	−0.007	−0.314	−0.065**	−2.144
Sports and Leisure Services	0.114***	5.081	0.132***	3.401	0.168***	2.962
Scientific and cultural services	0.132***	12.473	0.201***	11.485	0.225***	8.872
Cultural relics and historic sites	0.100***	10.229	0.201***	11.620	0.289***	11.340
Scenic spots	−0.069***	−5.532	−0.067***	−3.101	−0.039	−1.207
Yellow River related cultural facilities	−0.007**	−2.349	−0.016***	−2.880	−0.035***	−4.406

*represents the significance level; ***p < 0.01 indicates highly significant; ** 0.01 ≤ *p* < 0.05 indicates significant; * 0.05 ≤ *p* < 0.1 indicates weakly significant; *p* ≥ 0.1 indicates insignificant. The adjusted r-squared of the regression equation at the township travel circle level is 0.046; the adjusted r-squared of the regression equation at the county travel circle level is 0.041; and the adjusted *r*-squared of the regression equation at the city travel circle level is 0.034. All models additionally controlled for population size, distance to county seat, and distance to township center.

#### 3.2.2 Geographic weighted regression results

**Figure 7:**
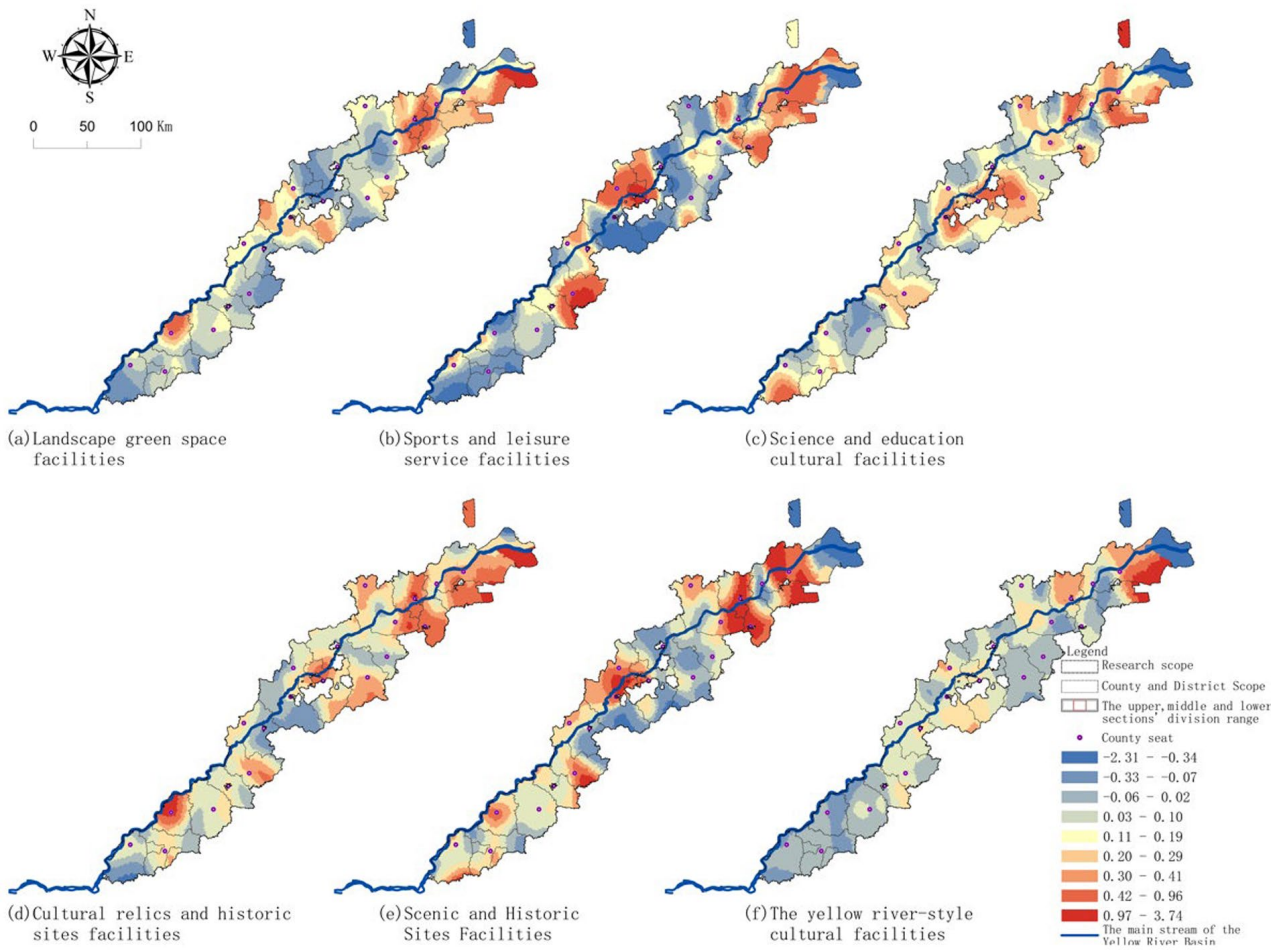
Geographically weighted regression results of township living circle: **(a)** landscape green space facilities; **(b)** sports and leisure service facilities; **(c)** science and education cultural facilities; **(d)** cultural relics and historic sites facilities; **(e)** scenic spots facilities; **(f)** Yellow River-related cultural facilities.

Due to the most significant spatial differences in the township living area, the influence intensity of the factors varies greatly among different regions, making it difficult for the OLS model to fully reflect this local spatial characteristic. Therefore, based on the multiple linear regression, this study introduces the geographically weighted regression model to conduct a further analysis of the spatial heterogeneity at the township scale and to present its spatial distribution characteristics. Figure 7 shows the results of the geographically weighted regression at the township scale. As shown in the figure, the color gradation reflects the strength and direction of the local influence, and a positive coefficient indicates that “the higher the accessibility, the larger the radius of the living area”.

Overall, various facilities along the line of Jinan—Dezhou—Zibo—Binzhou show generally high local coefficients, while the coefficients in the upstream areas such as Heze, Liaocheng, and some riverine areas are relatively low, presenting a spatial pattern of “significant in the middle section, high values concentrated, and greater differences between upstream and downstream”. Landscape greenery facilities (Figure 7a) form a slightly higher positive effect area around the central cities such as Jinan, Zibo, and Binzhou, while the coefficients in some upstream areas are relatively low, with overall differences but not extreme, presenting a moderate regional differentiation. Sports and leisure service facilities (Figure 7b) show a more obvious spatial gradient, with local coefficients significantly higher near the urban clusters in the middle section, forming a continuous high-value band, while some upstream areas are low-value zones, representing one of the facilities with the strongest spatial differentiation, and the response of rural residents to sports and leisure facilities varies significantly in different regions. Science and education cultural facilities (Figure 7c) also show higher spatial coefficients, especially in areas such as Jinan, Binzhou, and Heze, forming prominent high-value areas, indicating that this type of facility has the most obvious pulling effect on the radius of the living circle in the middle section. Compared with sports and leisure facilities, the high-value range of science and education cultural facilities is wider, belonging to a type with stronger cross-regional attractiveness and higher spatial heterogeneity. Cultural relic and ancient site facilities (Figure 7d) have point-like and patch-like clustering of local coefficients, represented by historical resource-intensive areas such as Tai’an, Heze, Binzhou, and Dongying, forming several relatively prominent high-value patches, while other areas are significantly lower, indicating that their influence shows a highly localized spatial difference. Scenic spots facilities (Figure 7e) have high-value areas in the Tai’an piedmont belt and the Dongying estuary coastal belt, while the corridor in the middle section is mostly at a low level, with significant coefficient differences. Yellow River culture facilities (Figure 7f) are the type with the smallest spatial variation and the weakest regional difference among the six types. The local coefficients in most areas remain at relatively close levels, with a smooth overall color band transition, and only slightly increase in the middle and lower reaches areas such as Binzhou—Dongying. This indicates that the influence of Yellow River culture nodes has strong spatial stability. In general, the spatial differences of sports and leisure, science and education cultural, and scenic spots facilities are the most prominent; cultural relic and ancient site facilities show a highly localized characteristic; while Yellow River culture facilities have the smallest spatial variation and the highest regional stability. This indicates that the response of rural residents to different types of cultural facilities varies significantly, depending not only on the attributes of the facilities themselves, but also on the joint influence of the urban system, road network, and natural conditions.

## 4 Discussion and conclusion

### 4.1 Discussion

The daily activities in rural areas are shaped by productive travel, social interactions, and seasonal participation, resulting in a living circle that is organizationally distinct from that of cities (19, 24). Based on the measurement of network distance, urban living circles are typically divided into three levels: neighborhood living circle (2–3 km), residential area living circle (4–6 km), and metropolitan living circle (12–20 km). Correspondingly, the three layers of rural living circles are township living circle (9–10 km), county living circle (14–15 km), and municipal living circle (16–30 km). It can be seen that the overall rural living radius is generally larger than that of cities. The median radii of the three corresponding scales are expanded by approximately 3–4 times. In terms of spatial distribution, smaller living radii are stably formed as continuous or semi-continuous belts around county seats and town seats, and increase outward along major roads. Outlying villages significantly expand due to insufficient road network distribution and low facility density (11). Crossing strong boundaries further elongates the effective distance. Taking the Yellow River region as an example, the sparse river-crossing passages make the “direct proximity” facilities on the opposite bank become farther in a network sense. Commonly, the additional detour distance increases by about 8–15 km, and cross-bank sharing thus converges more towards the internal circulation within the same bank. The living radius system in the boundary area systematically increases (25). Therefore, the scale and form of rural living circle are jointly determined by the county-town center system, road network structure, facility agglomeration and boundary connectivity.

In the rural travel structure, cultural landscape facilities are an important purpose, but their overall proportion is lower than that of productive and functional travel. Therefore, their role in influencing the scale of the living area is flexible and shows significant context dependence (3). From a behavioral perspective, cultural travel is mostly an optional activity, which is jointly regulated by time Windows and social networks (19). In daily life, it mainly relies on the green space, sports and science and education facilities in the core of the county and town. Festivals and tourism have triggered medium and long-distance travel across villages and even counties, driving up the travel radius of scenic spots and historical sites (26). From the perspective of the influence of the six types of cultural landscape facilities on the living area, landscape green spaces, sports and leisure, and science and culture facilities have a high spatial overlap with the county town center, short access paths, and certain substitutability, and are more stable in compressing the travel radius of the nearby area (27). Cultural relics and scenic spots are constrained by history and natural endowments, are scattered in distribution, and have limited traction for daily short-distance travel, but will significantly increase travel with larger radii during festivals and peak seasons. The Yellow River cultural facilities are distributed in series along the main stream, and the influence intensity varies with the continuity of the corridor and the connectivity conditions across the river. When the connection is smooth, the average reach distance of the coastal villages is significantly reduced, and the influence range Narrows rapidly when there is a break point. Overall, the effect of cultural landscape facilities on travel radius is not uniform, but is determined by the facility type, network structure, and season-event: in the core area of the county town, it shows a relatively small but stable change, while in the periphery and boundary areas, it shows a higher amplitude fluctuation.

The Yellow River serves as the root vein and collective memory carrier of regional culture. Cultural landscape facilities should be regarded as an important part of rural daily life. Their systematic construction holds substantial value for cultural inheritance and the cultivation of public nature, thus requiring a coordinated planning approach. Recent chrono-urbanism research further indicates that heritage preservation and community livability can be mutually reinforcing when heritage spaces are embedded in everyday life and supported by collaborative governance (28). Firstly, a hierarchical facility system should be constructed based on the scale of the living area (20). Village-level micro facilities cater to local and frequent needs, while township comprehensive facilities provide regular public cultural, sports and educational services. Corridor thematic facilities focus on the display, education and study tours related to the Yellow River theme, forming a structure that is interconnected in terms of service radius and function. Secondly, promote coordinated renewal of nodes, corridors and settlement surfaces (29). Using county-town centers and key towns as hubs, connect the top-of-the-bank pedestrian paths and interpretation systems with the rural road—branch road network, systematically repair broken points and weak links, and enhance the continuous accessibility and facility connection strength of the two wings of the countryside and the peripheral areas (30). Finally, for seasonal and event-related aspects, strengthen operation and content supply, forming an operation system based on regular activities. During the normal period, courses, performances and public reading are carried out relying on township comprehensive facilities and village-level micro facilities, maintaining the participation frequency. Integrate cultural relics, historical sites, scenic spots and special sites related to the Yellow River during festivals and agricultural seasons, organize thematic activities and routes, and enhance the intensity of collaborative use. Mobile units such as mobile books and touring performances are allocated to the outer edge and the restricted areas across the river to make up for the lack of accessibility and transform the event demand into relatively stable cultural acquisition.

It should be noted that there are also some limitations in this study. This data is based on active mobile phone signaling and may systematically underestimate the population without mobile phones or those using non-smart phones. In rural areas along the Yellow River with a high degree of aging, this bias is particularly significant. Therefore, the data field actually reflects the density of active mobile phone users, rather than the absolute population distribution. Additionally, the mobile phone signaling data used in the study is in a 500 m × 500 m grid, and there may be intermittent or missing records in signal-poor areas such as the riverbank beaches, resulting in the interruption or underestimation of the travel chains in remote villages, which may cause deviations in the true service range of cultural landscape facilities within remote living areas.

### 4.2 Conclusion

This study identified the three-level scale of the rural living circle along the Yellow River in Shandong Province. The median radii of the adjacent area—township—county/municipal area are approximately 8 km, 13 km, and 18 km, respectively. The low-value areas are stably distributed around the county and town centers and increase outward along the main roads. The outer edge shows a band-like increase along the river-crossing constraint zone. There are differences in the effects of the six types of cultural landscape facilities on the travel radius. Among them, landscape green spaces, sports and leisure facilities, and science and education cultural facilities are densely arranged in the core of the county and town, have short access paths, and can stably reduce the distance to the nearby area. Cultural relics and historical sites are constrained by history and natural endowments and are distributed sparsely, having limited influence on daily short-distance travel but extending long-distance travel during festivals and peak seasons. Yellow River-related facilities are connected along the main stream, and their effect intensity depends on the conditions of river-crossing passages. The MGWR results further reveal that the impact of accessibility on the living radius has significant spatial non-stationarity. The marginal effect in the central area is small and stable, while the outer edge and the river-crossing restricted area are more sensitive to the same amplitude improvement. In addition, different facilities had different effect scales, with the modern public class being more prominent at near-mesoscale, and the Yellow River class being more prominent at corridor and city scale. The local explanatory power was higher in the middle section where the corridor was continuous and the nodes were dense.

## Data Availability

The raw data supporting the conclusions of this article will be made available by the authors, without undue reservation.
